# O052. Migraine without aura and osteopathic medicine, a non-pharmacological approach to pain and quality of life: open pilot study

**DOI:** 10.1186/1129-2377-16-S1-A180

**Published:** 2015-09-28

**Authors:** Vito Adragna, Andrea S Bertino, Mauro Carano, Alessandro Soru, Giovanna Taranto, Riccardo Desideri

**Affiliations:** Research Department, Study Centre for Traditional Osteopathy, Rome, Italy

## Background

Migraine without aura is the most known and widespread primary headache, more than one person out of 10 suffers from this form. The management of the migraine patient is complex and can not be separated from a pharmacological approach, considering that alternative and complementary therapies are increasingly present in patient management [1, 2]. This study aimed to verify the efficacy of osteopathic manipulative treatment (OMT) in patients with migraine without aura.

## Methods

Eight subjects, three males and five females with migraine without aura (IHS: 1.1-ICD10:G43.0), selected at a private medical office, were included in a single treatment group. Four treatments were carried out in 8 weeks. Outcome measures were frequency of attacks, drug taking, MIDAS, HIT-6, SF-36 and BAQ (Body Awareness Questionnaire). Outcomes were measured at baseline (t_0_), 1 month after the last treatment (t_1_), and 3 months after the last treatment (t_2_), all subjects filled in a headache diary from three months before t_0_ and for the duration of the study and continued drug therapy prescribed.

## Results

**Figure 1:**
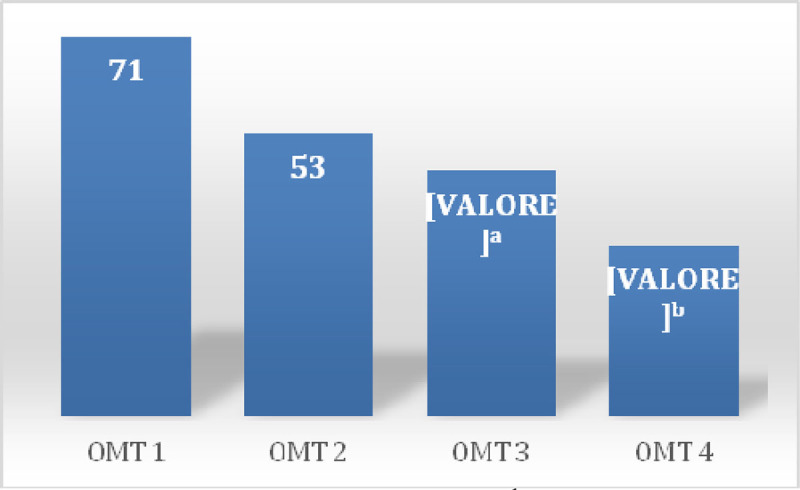
**Absolute frequency of total SD detected in the four OMT sessions**. ^a^t = 2.71 p = 0.01 CI = 0.61-5.62; ^b^t = 3.96 p = 0.001 CI = 2.21-7.52

**Figure 2:**
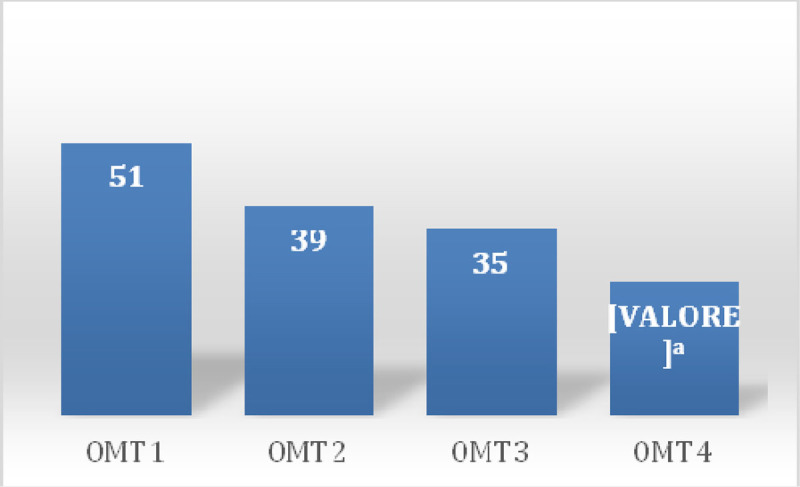
**Absolute frequency of Musculoskeletal SD detected in the four OMT sessions.**^a^t = 2.64 p = 0.02 CI = 0.59-5.90

**Figure 3:**
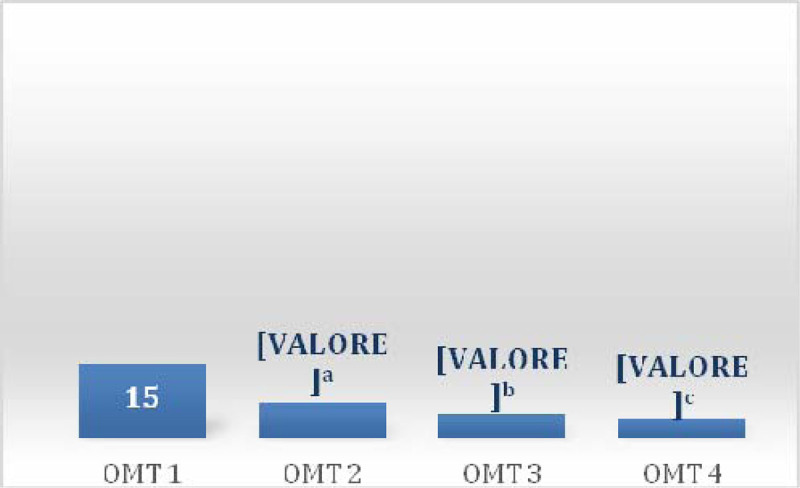
**Absolute frequency DS craniosacral system detected in the four OMT sessions**. ^a^t = 2.18 p = 0.04 CI = 0.02-1.97; ^b^t = 2.62 p = 0.02 CI = 0.22-2.27; ^c^t = 3.93 p = 0.001 CI = 0.61-2.12

**Figure 4:**
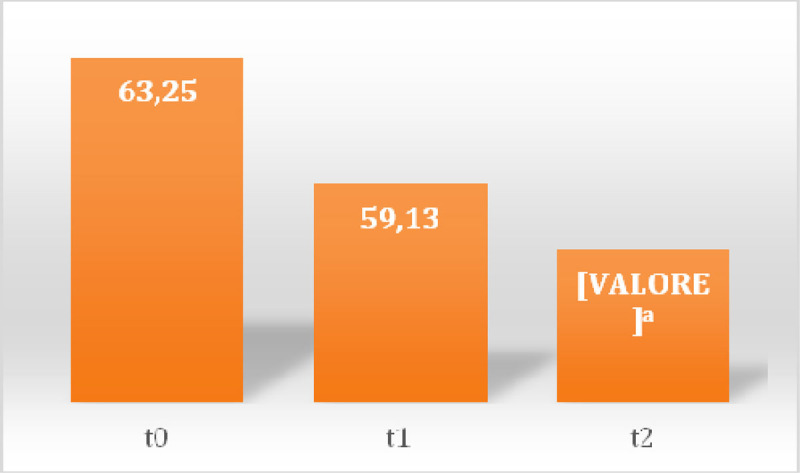
**Average scores of the HIT-6**. ^a^t = 2.13 p = 0.05 CI = 0.03-12.53

**Figure 5:**
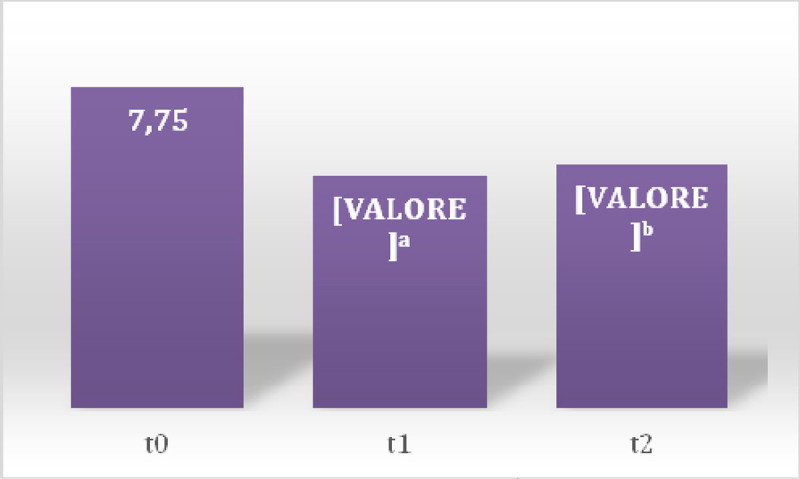
**Average scores of the MIDAS b**. ^a^t = 2.68 p = 0.01 CI = 0.41-3.84; ^b^t = 2.33 p = 0.03 CI = 0.15-3.6

**Figure 6:**
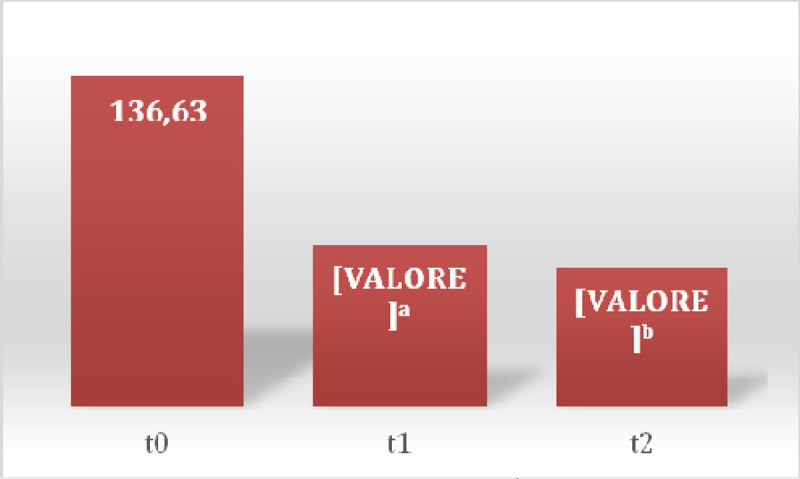
**Average scores of the SF-36**. ^a^t = 2.43 p = 0.02 CI = 1.6-25.39; ^b^t = 3.01 p = 0.01 CI = 4.26-26.47

**Figure 7:**
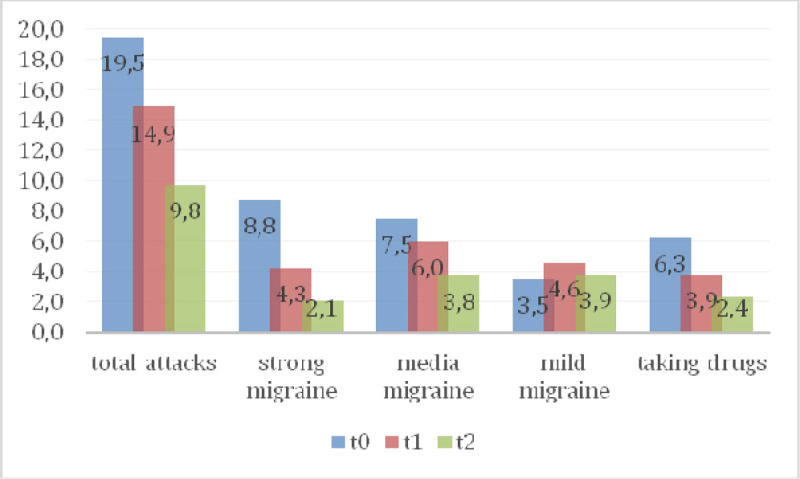
Absolute frequency in days of migraine attacks and taking drugs.

In the first session there was a prevalence of 100% of somatic dysfunctions (SD) in C1-occipital joint and in the other session a prevalence of 37% in the same joint (Table 1) was detected. Between sessions of OMT a reduction of SD was observed showing a significant reduction of total dysfunction at third (p = 0.01) and fourth (p = 0.001) treatment (Figure 1), the SD Musculoskeletal at fourth treatment (p = 0.02) (Figure 2) and those of the craniosacral system at the second (p = 0.04), the third (p = 0.02) and fourth (p = 0.001) treatment (Figure 3). Significant results were observed on the HIT-6 scale at t_2_ (p = 0.05) (Figure 4), MIDAS b scale score at t_1_ (p = 0.01) and t_2_ (p = 0.03) (Figure 5) and SF-36 scale at t_1_ (p = 0.02) and t_2_ (p = 0.01) (Figure 6). BAQ, the other item of MIDAS and the results of the headache diary, despite the reduction in the scores, did not produce significant results in the days of migraine attacks and medication taking (Figure 7).

**Table 1:** Prevalence (%) of somatic dysfunction per OMT session. Other dysfunctions have reported lower prevalences.

	Occ/C1	SBS^a^ compression	C^b^3	T^c^3	T^c^4	T^c^5	T^c^9	Sacrum
OMT 1	100	87						
OMT 2	37		37			37		37
OMT 3	37		37	37			37	
OMT 4	37		37		37			

^a^Spheno-Basilar Sincondrosy. ^b^Cervical vertebra. ^c^Thoracic vertebra.

## Conclusions

This study suggests that OMT has a positive effect on pain reduction and quality of life improvement in patients with migraine without aura. Future studies, contemplate including assessment of anxiety and depression, the use of a control group and follow-up in the long term.

Written informed consent to publish was obtained from the patient(s).
